# Effects of Combined Treatment with Acupuncture and Chunggan Formula in a Mouse Model of Parkinson's Disease

**DOI:** 10.1155/2019/3612587

**Published:** 2019-11-21

**Authors:** Tae-Yeon Hwang, Min-A Song, Sora Ahn, Ju-Young Oh, Dong-hee Kim, Quan Feng Liu, Wonwoong Lee, Jongki Hong, Songhee Jeon, Hi-Joon Park

**Affiliations:** ^1^Acupuncture and Meridian Science Research Center, Kyung Hee University, 26 Kyungheedae-ro, Dongdaemun-gu, Seoul 02447, Republic of Korea; ^2^Department of Korean Medical Science, Graduate School of Korean Medicine, Kyung Hee University, 26 Kyungheedae-ro, Dongdaemoon-gu, Seoul 02447, Republic of Korea; ^3^BK21 PLUS Korean Medicine Science Center, College of Korean Medicine, Kyung Hee University, 26 Kyungheedae-ro, Dongdaemoon-gu, Seoul 02447, Republic of Korea; ^4^Department of Meridian Medical Science, Graduate School of Korean Medicine, Kyung Hee University, 26 Kyungheedae-ro, Dongdaemoon-gu, Seoul 02447, Republic of Korea; ^5^Dongguk University Gyeongju, 123, Dongdae-ro, Gyeongju-si, Gyeongsangbuk-do, Gyeongju, Republic of Korea; ^6^College of Pharmacy, Kyung Hee University, 26 Kyungheedae-ro, Dongdaemoon-gu, Seoul 02447, Republic of Korea; ^7^Chonnam National University Medical School, 77, Yongbong-ro, Buk-gu, Gwangju 61186, Republic of Korea

## Abstract

Parkinson's disease is the second most common neurodegenerative disease. Patients with Parkinson's disease can be treated with a combination of acupuncture and herbal medicine, but studies on the synergistic effects of the combined treatment have not yet been conducted. Thus, we subjected an MPTP-induced Parkinson's disease mouse model to the combined treatment. We used acupoint GB34 for acupuncture and modified Chunggantang (KD5040) as the herbal medicine, as they have been reported to be effective in Parkinson's disease. We investigated the suboptimal dose of KD5040 and then used this dose in the combined treatment. The results showed that the combined treatment had a synergistic effect on improvements in abnormal motor function and neurodegeneration compared with the use of acupuncture or herbal medicine alone. The combined treatment also had a neuroprotective effect via the PI3K/AKT and MAPK/ERK signaling pathways. These findings suggest that the combined treatment with acupuncture and KD5040 can help improve the symptoms of Parkinson's disease.

## 1. Introduction

Parkinson's disease (PD) is a neurodegenerative disorder characterized by reduced dopamine secretion due to selective loss of dopaminergic neurons in the substantia nigra pars compacta (SNpc) [1, 2]. The clinical symptoms of PD are resting tremors, bradykinesia, rigidity, and instability of posture [3, 4]. The pathology of PD is related to numerous factors, such as oxidative stress, mitochondrial dysfunction, inflammation, and cell death [5–7]. There is no cure for PD, so the objective of treatments is to relieve the symptoms. Levodopa is the representative drug for improving the symptoms of PD. However, the dose of levodopa must be increased as treatment duration is extended, and more than 50% of patients with PD taking levodopa suffer from side effects, such as levodopa-induced dyskinesia (LID) [8, 9]. Therefore, fundamental treatments for PD patients are required [10–12].

Many studies have been conducted regarding the efficacy of acupuncture and herbal treatments for improving PD symptoms. The acupoint called “GB34” is the most effective acupuncture site [13]. Acupuncture treatment at GB34 activates the prefrontal cortex, precentral gyrus, and the putamen, which are the important brain regions for relieving PD symptoms [14]. Other researchers have reported that acupuncture at GB34 has a neuroprotective effect on dopaminergic neurons in a PD animal model [15]. It also enhances the availability of synaptic dopamine [16], decreases the effective dose of levodopa, and relieves side effects [17]. Thus, GB34 is an appropriate acupoint to treat PD [18–21].

As discussed by Bae et al. [22, 23], two herbal medicines have been shown to exhibit neuroprotective effects by enhancing autophagy. One study showed that herbal medicine is efficacious in improving the symptoms of drug-induced parkinsonism [24]. Other herbal medicines have a neuroprotective effect on dopaminergic neurons and improve motor symptoms by inhibiting oxidative stress and cell death [25] and by improving mitochondrial dysfunction [15]. Chunggantang is regarded as an efficient drug for improving the symptoms of PD. The modified form of Chunggantang (KD5040) is widely used in Traditional Oriental Medicine and has many effects, including anti-inflammatory and neuroprotective properties [26], improvement of motor function [27], decreasing the dose of levodopa, and relieving LID [28]. KD5040 also involved the neuronal survival in the brain by the expression of salusin-*β* and MrgprA1 [29].

Acupuncture and herbal medicine are used in combination rather than independently in clinical situation. However, in most studies, only the single-treatment effects of acupuncture and herbal medicine have been assessed. Thus, it is necessary to pragmatically evaluate the effects of a combined treatment with acupuncture and herbal medicine in PD.

In this study, acupuncture at GB34 and herbal medicine using KD5040 were selected as two therapies to treat PD. The objective of this study was to examine whether the treatment effects differed when the two therapies were used in combination.

## 2. Materials and Methods

### 2.1. Animals

C57BL/6 mice (9 weeks old, male, 21–25 g; Central Animal Laboratories Inc.) were used in the present study. All mice were housed in a constant temperature (24 ± 1°C) and constant humidity (60%) environment under a 12/12 h light/dark cycle with free access to food and water for 1 week before the experiment. All experiments were performed according to the criteria of Kyung Hee University Institutional Animal Care and Use Committee (KHUASP(SE)-14-052) and the recommendations of the National Institutes of Health and the Korean Academy of Medical Sciences.

### 2.2. Drug Treatment

**Figure 1:**
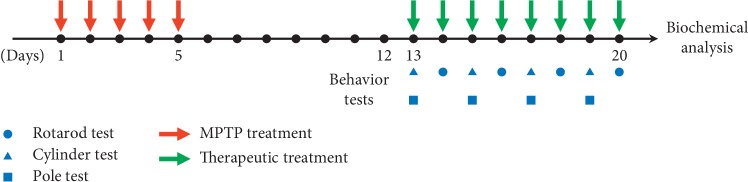
The experimental design. Saline or MPTP was administered to each mouse for 5 consecutive days. One week after the final administration, mice received one of three different treatments (acupuncture, KD5040, or the combined treatment). Behavioral tests were performed for 8 days.

Our study cited an experimental schedule of previous study [28]. Mice were injected with 1-methyl-4-phenyl-1,2,3,6-tetrahydropyridine (MPTP; 30 mg/kg, intraperitoneal for 5 days, Cayman Chemical, Ann Arbor, MI, USA). After administering MPTP and allowing 1 week for destruction of dopaminergic nerve fibers and dopaminergic neurons, two doses of KD5040 (low and high doses) were administered for 8 days. KD5040 was included in the food taken by the animals (Figure 1).

### 2.3. Animal Groups

To investigate the suboptimal dose of KD5040, the mice were divided into four groups as follows: control group (saline injection, C group, *n* = 8), MPTP group (MPTP injection, M group, *n* = 8), low-dose KD5040 (MPTP injection + 100 mg/kg KD5040, M + KDL group, *n* = 8), and high-dose KD5040 (MPTP injection + 300 mg/kg, M + KDH group, *n* = 8).

Then, we observed the synergistic effect between the acupuncture and herbal medicine treatment using a suboptimal dose of KD5040. The mice were divided into five groups as follows: control group (saline injection, C group, *n* = 8), MPTP group (MPTP injection, M group, *n* = 8), acupuncture group (MPTP injection + acupuncture at GB34, M + Acu group, *n* = 8), low-dose KD5040 (MPTP injection + 100 mg/kg KD5040, M + KDL group, *n* = 8), and the combined treatment group (MPTP injection + acupuncture at GB34 + 100 mg/kg KD5040, M + Acu + KDL group, *n* = 8). All experimental groups were randomly assigned to each group after the 1 week induction time.

### 2.4. Acupuncture Treatment

The acupuncture treatment was performed once a day for 8 days using acupuncture needles (0.20 mm diameter × 15 mm length, stainless steel, Haeng-lim-seo-weon Acuneedle Co., Gyeonggi-do, Korea) at GB34. The anatomical location of GB34 is an anterior and distal concave point from the head of the fibula. Mice were restrained tightly, and the needle was inserted at a depth of 3 mm from the skin and twisted twice per second for 30 s and then removed. To minimize stress on the mice, the acupuncture treatment is done accurately and quickly. The control and MPTP groups also received this method to give the same stress stimulus.

### 2.5. KD5040 Production

The method producing KD5040 is the same as the previous study [28]. The Chunggantang extract was composed of the rhizome of *Paeonia lactiflora* (29.41 g), rhizome of *Ligusticum chuanxiong* (19.61 g), rhizome of *Angelica gigas* (19.61 g), rhizome of *Bupleurum falcatum* (15.69 g), fructus of *Gardenia jasminoides* (7.84 g), and rhizome of *Paeonia suffruticosa* (7.84 g). They were pulverized and extracted twice with 10 vol. of 30% ethanol at 100°C with a reflux condenser for 3 h and then lyophilized after filtering with a 50 *μ*m filter. To strengthen the curative effect, flowers of *Eugenia caryophyllata* (100 g) and *Pogostemon cablin* (100 g) were prepared as described above. All medicinal herbs were provided by Dongguk University Medical Center. KD5040 comprised a 3 : 1 : 1 ratio of Chunggantang, *Eugenia caryophyllata*, and *Pogostemon cablin* and stored at −80°C (deposit #: KHH-G-0054).

### 2.6. Motor Function Test

Three tests were performed: the rotarod test, the cylinder test, and the pole test. The rotarod test (MED Associates Inc., St. Albans, VT, USA) was performed to measure neurological damage and evaluates coordinated function of limb movement and sense of balance. The duration of mice running on the rod was measured in seconds, and the maximum time was 480 s. The velocity of the spinning rod was increased gradually from 0 to 35 rpm. In the cylinder test, mice were placed in a plastic cylinder (12 cm diameter, 20 cm height) and filmed for 5 min. The number of times the mouse held the wall with their front limbs was counted. The pole test was used to evaluate bradykinesia in the PD mouse model. The pole was 55 cm height and 1.3 cm diameter. The mice were placed on the top of the pole, and the time that the mice turned around and reached the bottom was measured in seconds.

### 2.7. Immunohistochemistry

Mice were anesthetized using ether and perfused with 50 mM phosphate-buffered saline (PBS) for 5 min through the left ventricle. Then, 4% paraformaldehyde (PFA) in 0.2 M phosphate buffer was perfused for 10 min. After perfusion, the brain was extracted and stored in 4% PFA solution (4°C) for 24 h. The fixed brain was dehydrated in a 30% sucrose solution and sectioned at 40 *μ*m thickness using a freezing microtome (Leica, Nussloch, Germany). The brain sections were washed in 50 mM PBS and activated in 1% H_2_O_2_ to remove peroxidase for 15 min. Then, the sections were washed again and blocked in a solution which comprised 0.3% BSA and 3% Triton X-100 for 1 h. The striatum was activated using rabbit antityrosine hydroxylase antibody (TH, 1 : 1000, Santa Cruz Biotechnology, Santa Cruz, CA, USA; sc-14007) at room temperature for 24 h. The SNpc was activated using anti-TH antibody (1 : 4000) at 4°C for 72 h. The brain sections were washed again and activated using avidin-biotinylated peroxidase complex (Vectastain Elite ABC kit, Vector Laboratories, Burlingame, CA, USA) solution at room temperature for 1 h. After activation, the brain sections were developed using 3,3′-diaminobenzidine tetrahydrochloride (0.02% diluted with 50 mM Tris-buffer, pH 7.6), after adding 0.02% H_2_O_2_ for 1 min and 30 s. The developed sections were attached to slides coated with gelatin and dehydrated in 70, 80, 90, and 100% ethanol. The slides were cleared in xylene and mounted using Permount (Synthetic Mountant, Thermo Scientific, Waltham, MA, USA). The histological images of the striatum and SNpc were obtained by using a bright-field microscope (BX53, Olympus Japan Co., Tokyo, Japan). The optical density (OD; % of C group) value of the striatum dyed with TH was measured using Scion image (Scion Co., Frederick, MD, USA), and TH-positive dopaminergic neurons in the SNpc were counted. TH-positive neurons were counted manually. The SNpc region was selected based on an atlas of the mouse brain: the region between AP −3.08 and −3.28 mm from the bregma in the midbrain [30]. During analyses, observers were blinded to the experimental conditions to prevent the risk of observer bias.

### 2.8. Western Blot

Brain samples were homogenized in 200 *μ*l of lysis buffer (CyQUANT; Invitrogen, Eugene, OR, USA) including phosphatase inhibitor and protease inhibitor cocktail tablets. After homogenization, the samples were centrifuged at 12,000 rpm for 15 min at 4°C, and the supernatants were collected. The amount of protein was measured using the BCA assay. Quantified protein samples (10 *μ*g) were separated by 4–12% sodium dodecyl sulfate-polyacrylamide gradient gel (Invitrogen) electrophoresis and transferred to PVDF membranes (Immobilon-P, Merck Millipore Ltd., Darmstadt, Germany). The membranes were washed in tris-buffered saline with 0.1% Tween 20 (TBS-T) three times for 10 min and then blocked in 5% skim milk for 1 h. After blocking, the membranes were activated with antibodies to brain-derived neurotrophic factor (BDNF, 1 : 200, Santa Cruz Biotechnology; sc-546), TH (1 : 3000, Santa Cruz Biotechnology, sc-14007), *β*-actin (1 : 40,000, Sigma-Aldrich, St. Louis, MO, USA; A1978), phospho-inhibitory kappa B alpha (pI*κ*B*α* Ser32, 1 : 500, Cell Signaling Technology, Beverly, MA, USA; #2859), I*κ*B*α* (1 : 500, Cell Signaling Technology, #4814), phospho-extracellular signal-regulated kinase (pERK Thr202/Tyr204, 1 : 500, Cell Signaling Technology; #4370), ERK (1 : 500, Cell Signaling Technology, #9102), phospho-protein kinase B (pAKT Ser473, 1 : 1000, Cell Signaling Technology, #4058), AKT (1 : 1000, Cell Signaling Technology, #4691), phospho-cAMP response element-binding protein (pCREB Ser133, 1 : 200, Cell Signaling Technology, #9198), CREB (1 : 500, Cell Signaling Technology, #9197), phospho-glycogen synthase kinase 3 beta (pGSK3*β* Ser9, 1 : 250, Cell Signaling Technology, #9336), and GSK3*β* (1 : 200, Cell Signaling Technology, #9315) overnight at 4°C. The activated membranes were washed in TBS-T and activated with secondary horseradish peroxidase-conjugated goat anti-rabbit antibody (Pierce, Rockford, IL, USA) or mouse (Thermo Scientific, Waltham, MA, USA; PA1-30355) antibodies for 1 h. The membranes were washed again with TBS-T, and the proteins attached to the membranes were visualized using an enhanced chemiluminescence system (Pierce ECL Western Blotting Substrate, Thermo, Grand Island, NY, USA). OD values of the visualized protein bands were measured using ImageJ software (National Institutes of Health, Bethesda, MD, USA).

### 2.9. Cell Culture and Viability Test

SH-SY5Y cells, which were from a human neuroblastoma cell line, were obtained from the American Type Culture Collection (Manassas, USA) and maintained in Dulbecco's Modified Eagle's Medium containing 10% fetal bovine serum (Sigma-Aldrich, USA) and 1% antibiotics (GE Healthcare Life Sciences, USA). The cells were cultured in a humidified atmosphere of 5% CO_2_ and 95% air at 37°C. The media were replaced every 2 days. To investigate the cytotoxicity of the KD5040 extract on cell viability, the cells were cultured with the indicated concentrations for 24 h. The treated cells were incubated with 2 mg/mL of 3-(4,5-dimethylthiazol-2-yl)-2,5-diphenyltetrazolium bromide (MTT, Sigma-Aldrich, USA) at 37°C in a CO_2_ incubator for 4 h. Then, the MTT medium was carefully aspirated, and the formazan dye was eluted with dimethyl sulfoxide. The absorbance was measured with a spectrophotometer (Versamax; Molecular Devices, USA) at a wavelength of 580 nm.

### 2.10. Statistical Analysis

GraphPad Prism 5 (GraphPad Software, Inc., San Diego, CA, USA) was used to determine mean ± standard error of all of the data. One-way analysis of variance (ANOVA) was performed to compare the mean values of three or more groups, and the Newman–Keuls *post hoc* test was used to compare differences between experimental groups if the values were significant. A *p* value <0.05 was considered significant.

## 3. Results

### 3.1. Effect of Two Doses of KD5040

Before researching the synergistic effect of the combined acupuncture and KD5040 treatment, the effect of different doses (100 and 300 mg/kg) of KD5040 was compared in the PD induced mouse model.

#### 3.1.1. Effect of KD5040 on Motor Function Tests to Determine the Suboptimal Dose

**Figure 2:**
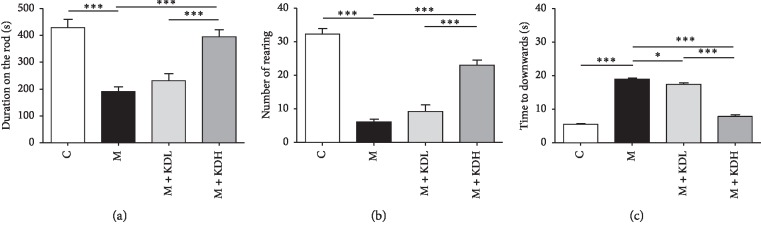
The motor function test revealed that significant recovery occurred on day 20 in the MPTP group with KD5040, compared with the MPTP group. Results of motor function tests in the four groups: (a) rotarod test, (b) cylinder test, and (c) pole test. ^*∗*^*p* < 0.05 and ^*∗∗∗*^*p* < 0.001 by one-way ANOVA followed by the Newman–Keuls *post hoc* test (*n* = 8–10 for each group).

One-way ANOVA of the rotarod test showed significant differences among the whole groups (*F*_3, 30_ = 22.56, *p* < 0.0001). The duration on the spinning rod decreased significantly in the M group compared with the C group (*p* < 0.001). The duration of the M + KDL group was not different from that of the MPTP group, but the duration of the M + KDH group was significantly higher than that of the M group (*p* < 0.001) (Figure 2(a)).

One-way ANOVA was performed to analyze results on cylinder test (*F*_3, 30_ = 67.21, *p* < 0.0001). The number of times in the M group was decreased compared with the number of times in the C group (*p* < 0.001). The number of times in the M + KDL group increased slightly, but the number of times in the M + KDH group increased significantly compared with the M group (*p* < 0.001) (Figure 2(b)).

Statistical analysis on pole test revealed a significant difference among the whole experimental groups [*F*_3, 28_ = 257.7, *p* < 0.0001]. The M group took more time to travel from the top to the bottom than the C group (*p* < 0.001). The time of the M + KDL group was slightly different than the time of the M group (*p* < 0.05), but the time of the M + KDH group decreased significantly compared to that of the M group (*p* < 0.001) (Figure 2(c)).

#### 3.1.2. State of Dopaminergic Nerve Fibers and Dopaminergic Neurons in the Striatum and SNpc

A histological analysis was performed to determine the effect of KD5040 on MPTP neurotoxicity. MPTP largely induced a loss of dopaminergic neurons after investigating dopaminergic nerve fibers in the striatum and SNpc.

**Figure 3:**
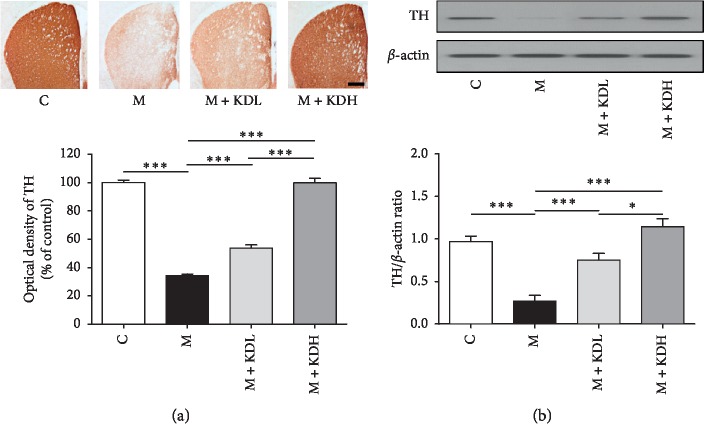
Tyrosine hydroxylase (TH) immunoreactivity and TH expression in the striatum. (a) TH-positive dopaminergic nerve fibers in the striatum (scale bar; 200 *μ*m) and optical density of TH-positive dopaminergic nerve fibers in the striatum. (b) Expression of TH in the striatum by western blot and quantification of TH expression relative to *β*-actin for each group. ^*∗*^*p* < 0.05 and ^*∗∗∗*^*p* < 0.001 by one-way ANOVA followed by the Newman–Keuls *post hoc* test (*n* = 4 for each group).

**Figure 4:**
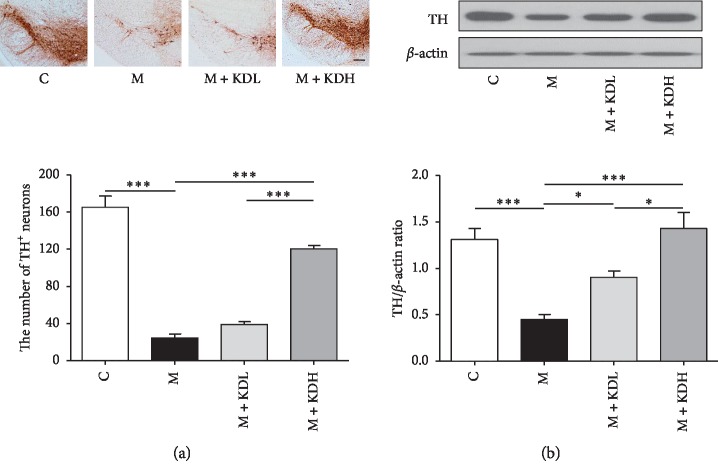
TH immunoreactivity and TH expression in the substantia nigra pars compacta (SNpc). (a) The number of TH-positive dopaminergic neurons in the SNpc (scale bar; 100 *μ*m) and total TH-positive cell number counted in the SNpc. (b) Expression of TH in SNpc by western blot and quantification of TH expression relative to *β*-actin on the striatal sides for each group. ^*∗*^*p* < 0.05 and ^*∗∗∗*^*p* < 0.001 by one-way ANOVA test followed by Newman–Keuls *post hoc* test (*n* = 4 for each group).

One-way ANOVA showed a significant difference on dopaminergic nerve fibers in the striatum (*F*_3, 12_ = 220.4, *p* < 0.0001) and dopaminergic neurons in SNpc (*F*_3, 12_ = 97.65, *p* < 0.0001). Dopaminergic nerve fibers in the M + KDL group increased significantly compared to those in the M group (*p* < 0.001) and those in the M + KDH group recovered to the level of the C group (*p* < 0.001) (Figure 3(a)). The number of dopaminergic neurons in the M group decreased significantly compared with the number in the C group in the SNpc 20 days after MPTP treatment (*p* < 0.001). The number in the M + KDL group increased slightly (*p* < 0.01), and the number in the M + KDH group increased significantly (*p* < 0.001 vs. M group; Figure 4(a)).

Statistical analysis of the TH expression on the western blot revealed that there was a significant difference in the striatum (*F*_3, 12_ = 315.0, *p* < 0.0001) and SNpc (*F*_3, 12_ = 5541, *p* < 0.0001) among whole groups. The TH expression of the M + KDH group is higher than that of the control group, but there is no significant difference in both striatum and SNpc. The TH expression in the M group decreased significantly in both regions compared with those in the C group (*p* < 0.001). The TH expression in the M + KDL and the M + KDH groups showed a significant increase in both two regions relative to those in the M group (*p* < 0.001) (Figures 3(b) and 4(b)). These results indicate a dose-dependent effect of KD5040. High-dose KD5040 had a greater neuroprotective effect than low-dose KD5040, so it was inappropriate to use high-dose KD5040 investigating the synergistic effect of the combined treatment. Therefore, the M + KDL group was selected for researching the synergistic effects with acupuncture treatment.

### 3.2. Synergistic Effects of the Combined Treatment of Acupuncture and KD5040 on Motor Function Tests in the MPTP-Induced PD Mouse Model

#### 3.2.1. Rotarod Test

**Figure 5:**
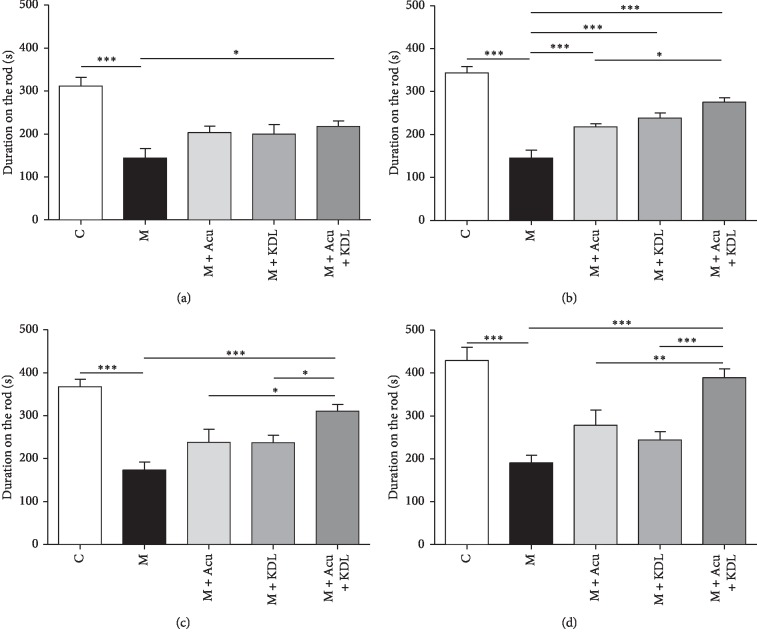
Synergistic effects of the combined treatment on time spent on the rod during the rotarod test. The rotarod was tested on (a) day 2, (b) day 4, (c) day 6, and (d) day 8. ^*∗*^*p* < 0.05, ^*∗∗*^*p* < 0.01, and ^*∗∗∗*^*p* < 0.001 by one-way ANOVA followed by the Newman–Keuls *post hoc* test (*n* = 8–10 for each group).

One-way ANOVA of the rotarod test showed a significantly different duration on the rod among the groups as day 2 (*F*_4, 37_ = 10.13, *p* < 0.0001), day 4 (*F*_4, 37_ = 29.44, *p* < 0.0001), day 6 (*F*_4, 37_ = 13.90, *p* < 0.0001), and day 8 (*F*_4, 37_ = 15.73, *p* < 0.0001). The duration in the M group decreased largely on the second day of the test compared with the time in the C group, and this result was consistent for 8 days (*p* < 0.001). The time in the M + Acu + KDL group increased significantly compared with the M group over the 8 days (*p* < 0.001), but there was a slight or no increase in time in the M + KDL and M + Acu groups (Figure 5).

#### 3.2.2. Cylinder Test

**Figure 6:**
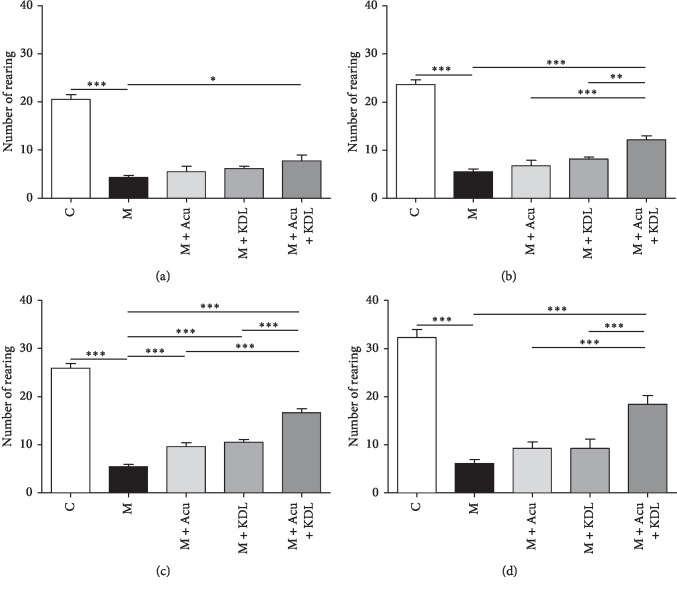
Synergistic effects of the combined treatment on number of rearing in the cylinder test. (a) Day 1, (b) day 3, (c) day 5 and (d) day 7. ^*∗*^*p* < 0.05, ^*∗∗*^*p* < 0.01, and ^*∗∗∗*^*p* < 0.001 by one-way ANOVA followed by the Newman–Keuls *post hoc* test (*n* = 8–10 for each group).

Statistical analysis of cylinder test revealed that a number of rearing showed significant difference among the whole groups as day 1 (*F*_4, 37_ = 56.70, *p* < 0.0001), day 3 (*F*_4, 37_ = 81.69, *p* < 0.0001), day 5 (*F*_4, 37_ = 109.8, *p* < 0.0001), and day 7 (*F*_4, 37_ = 47.12, *p* < 0.0001). The results were similar to the rotarod test. On the whole days of the test, the number of times rearing the wall in the M group decreased significantly compared with the number in the C group (*p* < 0.001). The number in the M + Acu and M + KDL groups showed no significant increase compared with the M group. However, the number in the M + Acu + KDL group increased significantly than the number in the M group after 7 days (*p* < 0.001) (Figure 6).

#### 3.2.3. Pole Test

**Figure 7:**
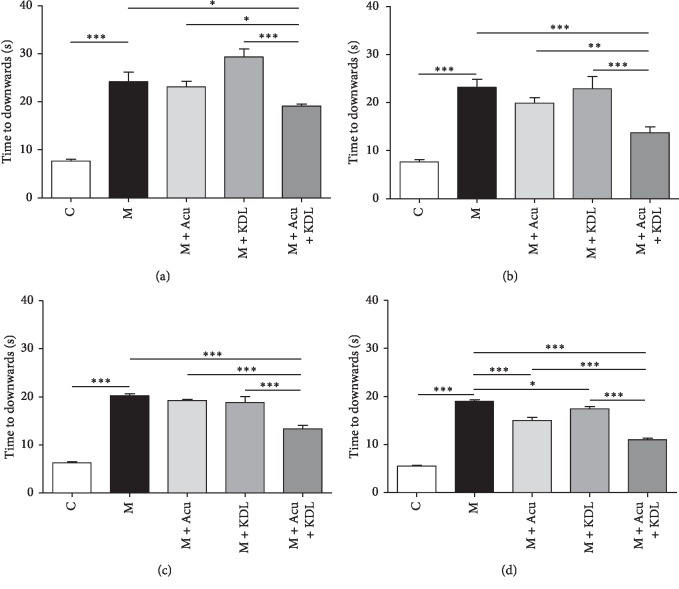
Synergistic effects of the combined treatment on time spent getting from the top of the pole to the floor on the pole test. (a) Day 1, (b) day 3, (c) day 5, and (d) day 7. ^*∗*^*p* < 0.05, ^*∗∗*^*p* < 0.01, and ^*∗∗∗*^*p* < 0.001 by one-way ANOVA followed by the Newman–Keuls *post hoc* test (*n* = 8–10 for each group).

One-way ANOVA showed a significant difference among the whole groups as day 1 (*F*_4, 35_ = 39.17, *p* < 0.0001), day 3 (*F*_4, 35_ = 18.22, *p* < 0.0001), day 5 (*F*_4, 35_ = 73.03, *p* < 0.0001), and day 7 (*F*_4, 35_ = 151.7, *p* < 0.0001) on the pole test. The M, M + Acu, and M + KDL groups showed bradykinesia on the first day, which was not observed in the C group after analyzing the time from top to bottom (*p* < 0.001). After 7 days, the time in the M + KDL group decreased significantly (*p* < 0.05), and a more significant decrease was detected in the M + Acu group (*p* < 0.001) compared with the M group. Moreover, the M + Acu + KDL group showed a better treatment effect than the single treatment groups, suggesting a synergistic effect of the combined treatment (*p* < 0.001) (Figure 7).

### 3.3. Effect of the Combined Treatment on Dopaminergic Neuron Loss Induced by MPTP

**Figure 8:**
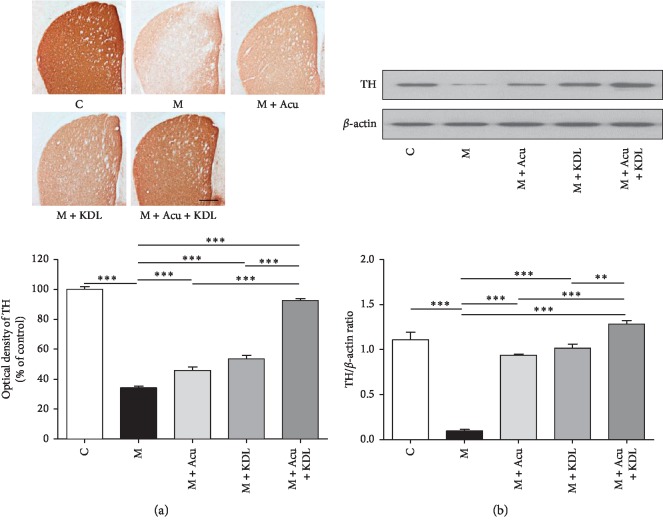
Combined treatment ameliorates MPTP-induced dopaminergic neurodegeneration in the striatum. (a) Immunohistochemistry of TH-positive dopaminergic nerve fibers in the striatum (scale bar; 200 *μ*m) and optical density of TH-positive dopaminergic nerve fibers in the striatum. (b) Expression of TH in the striatum by western blot and quantification of TH expression relative to *β*-actin for each group. ^*∗∗*^*p* < 0.01 and ^*∗∗∗*^*p* < 0.001 by one-way ANOVA followed by the Newman–Keuls *post hoc* test (*n* = 4 for each group).

**Figure 9:**
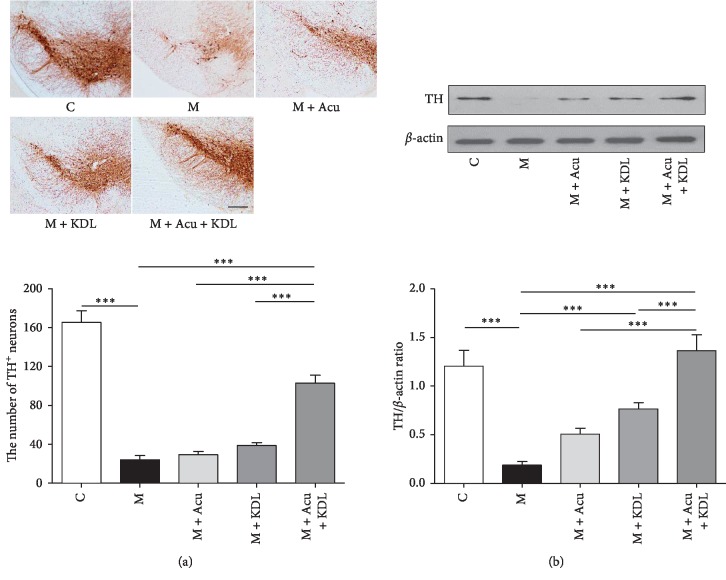
Combined treatment ameliorates MPTP-induced dopaminergic neuron degeneration in the SNpc. (a) Number of TH-positive dopaminergic neurons in the SNpc (scale bar; 100 *μ*m) and total TH-positive cell number counted in the SNpc. (b) Expression of TH in the SNpc by western blot and quantification of TH expression relative to *β*-actin on each striatal side for each group. ^*∗∗∗*^*p* < 0.001 by one-way ANOVA followed by the Newman–Keuls *post hoc* test (*n* = 4 for each group).

To quantify TH-positive cells, we measured the OD values of TH-positive dopaminergic nerve fibers in the striatum (Figure 8) and counted the number of TH-positive dopaminergic neurons in the SNpc (Figure 9).

One-way ANOVA of the OD value in the striatum (*F*_4, 15_ = 257.5, *p* < 0.0001) and the number of TH-positive dopaminergic neurons in the SNpc (*F*_4, 15_ = 73.40, *p* < 0.0001) showed a significant difference among the whole experimental groups. The OD value in the M group decreased significantly compared to that in the C group (*p* < 0.001). The OD values in the M + Acu, M + KDL, and M + Acu + KDL groups increased significantly compared with the M group (*p* < 0.001) (Figure 8(a)). The number of neurons in the M group largely decreased compared to that in the C group (*p* < 0.001). The number in the M + Acu and M + KDL groups had no significant difference, and the number in the M + Acu + KDL group increased significantly compared to that in the M group (*p* < 0.001) (Figure 9(a)).

Statistical analysis of the TH expression on the western blot revealed a significant difference among the whole groups in the striatum (*F*_4, 15_ = 230.0, *p* < 0.0001) and SNpc (*F*_4, 15_ = 3494, *p* < 0.0001). The loss of TH protein induced by MPTP in the M group decreased largely compared with the C group in both striatum and SNpc (*p* < 0.001). The loss of TH protein was inhibited by both single and combined treatments, and that of the M + Acu + KDL group was inhibited largely compared with other groups (*p* < 0.001) (Figures 8(b) and 9(b)).

### 3.4. Effect of the Combined Treatment on the Expression of Proteins Related to the Neuroprotective Effect

**Figure 10:**
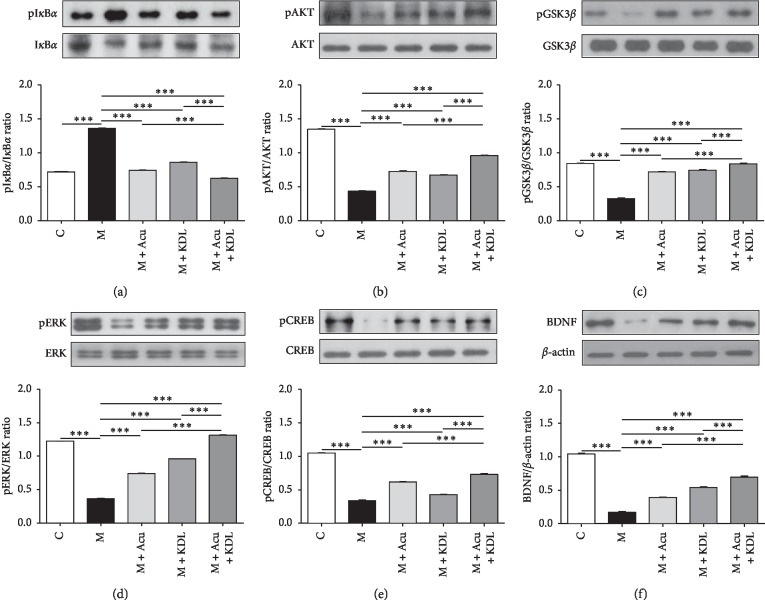
Protective effects of the combined treatment in Parkinson's disease mouse model. The SNpc was isolated and immunoblotted with the indicated antibodies. The intensities of the protein bands were quantitated by densitometry, and the phosphorylated forms were normalized to the total form or *β*-actin. Changes in (a) pI*κ*B*α*, (b) pAKT, (c) pGSK3*β*, (d) pERK, (e) pCREB, and (f) BDNF. Data are mean ± standard error. ^*∗∗∗*^*p* < 0.001 by one-way ANOVA followed by the Newman–Keuls *post hoc* test (*n* = 4 for each group).

Western blotting was performed to investigate the effect of the combined treatment on the expression of six proteins. One-way ANOVA of the ratios of pI*κ*B*α*/I*κ*B*α* (*F*_4, 15_ = 2889, *p* < 0.0001), pAKT/AKT (*F*_4, 15_ = 1721, *p* < 0.0001), pGSK3*β*/GSK3*β* (*F*_4, 15_ = 674.5, *p* < 0.0001), pERK/ERK (*F*_4, 15_ = 6852, *p* < 0.0001), pCREB/CREB (*F*_4, 15_ = 728.3, *p* < 0.0001), and BDNF/*β*-actin (*F*_4, 15_ = 591.9, *p* < 0.0001) were performed and showed a significant effect of the combined treatment. Expression of pI*κ*B*α* increased in the M group compared with the C group (*p* < 0.001). The increase in the M group clearly decreased in the M + Acu and M + KDL groups, and a greater decrease in pI*κ*B*α* expression was observed in the M + Acu + KDL group (*p* < 0.001) (Figure 10(a)). Expression of pAKT, pGSK3*β*, pERK, pCREB, and BDNF decreased in the M group compared with the C group (*p* < 0.001). All of these proteins were increased significantly in the single treatment groups compared with the M group (*p* < 0.001), and significant increases were observed in the M + Acu + KDL group compared with the M, M + Acu, and M + KDL groups (*p* < 0.001) (Figures 10(b)–10(f)).

## 4. Discussion

In this study, we investigated the synergistic effect of the combined treatment of acupuncture at GB34 and KD5040. We found that the combined treatment had a synergistic effect on motor function enhancement and a neuroprotective effect on dopaminergic neurons compared with the use of acupuncture or KD5040 alone.

We preferentially performed the tests using a suboptimal dose of KD5040, which has a lower dose than generally used clinically, to detect the synergistic effect of the combined treatment. We confirmed that Acu or KD5040 alone did not affect to the motor function of control mice ([Supplementary-material supplementary-material-1] in the Supplementary Materials). Treatment with acupuncture or KD5040 alone did not enhance motor function compared with that of the M group, but motor dysfunction was significantly alleviated in the M + Acu + KDL group. Moreover, the OD values of dopaminergic nerve fibers in the striatum and the number of dopaminergic neurons in the SNpc increased significantly in the M + Acu + KDL group compared to those in the M group, indicating that degeneration of dopaminergic neurons recovered in response to the combined treatment.

To determine how the combined treatment exhibited a neuroprotective effect, we investigated changes in neuroprotection-related protein expression in the SNpc by western blot. pI*κ*B*α*, which increased in response to MPTP, decreased in the M + Acu and M + KDL groups, and particularly in the M + Acu + KDL group. Neural inflammation plays a crucial role in neurodegenerative processes. Nuclear factor-kappa B (NF*κ*B) has been proposed as a target for controlling neuronal death and acute or chronic inflammation [31]. Inactive I*κ*B*α* combines with NF*κ*B to inhibit NF*κ*B activity, but when I*κ*B*α* is phosphorylated, the combination of I*κ*B*α* and NF*κ*B is broken and NF*κ*B is activated [32]. As shown in this study, activation of NF*κ*B caused by increased pI*κ*B*α* was reduced to the level of the C group by the combined treatment. This result indicates that the combined treatment suppressed the death of dopaminergic neurons by inhibiting phosphorylation of I*κ*B*α*. The PI3K/AKT pathway is a representative signaling pathway known to protect nerve cells from extracellular injury [33]. AKT has been linked to a variety of cell functions, such as survival, growth, death, differentiation, transcription, and migration and has a neuroprotective effect on dopaminergic neurons [34]. The expression of inflammatory cytokines increases when phosphorylation of AKT is suppressed, and this is known to increase the inflammatory response in a brain damage state [35]. Lin et al. [20] reported that the electroacupuncture treatment at GB34 and LR3 had neuroprotective effects modulating BDNF and AKT pathways. Administration of MPTP inhibited phosphorylation of AKT in the striatum and SNpc, whereas the combined treatment normalized the level of AKT phosphorylation. Activation of GSK3*β* is related to cell death in PD [36]. The results of this study indicate that the combined treatment activated the PI3K/AKT/GSK3*β* pathway in dopaminergic neurons. The MEK/ERK pathway has been reported to regulate cell proliferation, differentiation, survival, and death and to play a crucial role in PD. Activation of ERK activates pCREB and increases survival of dopaminergic neurons [37, 38], whereas inhibiting the MEK/ERK pathway induces cell death and suppresses proliferation by stimulating both the intrinsic and extrinsic apoptosis pathways [39]. Phosphorylation of ERK was suppressed by MPTP treatment, but activation of ERK increased significantly in response to the combined treatment, suggesting that the MEK/ERK pathway is involved in the neuroprotective effect of the combined treatment. CREB regulates the expression of gene-encoding proteins involved in regulating important brain functions, such as generation of neurons, learning, memory, metabolism, and neuroplasticity [40, 41]. BDNF mediates cell generation, neuroplasticity, cell proliferation, and nerve survival [42, 43]. Phosphorylation of CREB, a marker of neuronal activity, induces many genes including BDNF, and we found that the combined treatment increased pCREB and BDNF levels. The combined treatment has antiapoptosis and neuroprotective effects on dopaminergic cells [44–46].

Patients with PD must take medication for their entire life. To ensure that the long-term administration of drugs has therapeutic effects, a patient's gastrointestinal system must function normally. However, most PD patients experience gastrointestinal disorders (GIDs) including dysphagia, constipation, and gastroesophageal reflux [47]. These gastrointestinal systems may be negatively affected by taking the high-doses of medications over a long period. Therefore, it is essential to develop efficient treatments that minimize the burden on the gastrointestinal system.

The present study demonstrated that the high-dose KD5040 has the significant therapeutic effect. However, the focus of our study is not the therapeutic effect of high-dose KD5040, but the synergistic effect between KD5040 and acupuncture treatment. We regarded that the high-dose KD5040 was insufficient to confirm the combined treatment for researching the synergistic effect because the M + KDH group showed similar results to the control group. High-dose KD5040 also led to cytotoxicity ([Supplementary-material supplementary-material-1] in the Supplementary Materials). The significant toxic effect of KD5040 was observed at doses above 200 mg/kg. Mice treated with a dose of 100 mg/kg of KD5040 alone did not exhibit significant differences compared with the control group, so we tried to evaluate the synergistic effect by choosing this low-dose KD5040 which is suboptimal and has a neuroprotective effect.

A previous study reported that acupuncture normalized abnormal motor function by increasing the availability of reduced dopamine [16]. Additionally, acupuncture treatment increased the efficiency of levodopa at doses lower than those normally considered effective. Acupuncture treatment also reduced the side effects of levodopa [17]. Together, these results suggest that the combination of acupuncture and herbal medicines may have synergistic effects. Based on these findings, we explored the synergistic effects of the combined treatment using a suboptimal dose of KD5040 and acupuncture.

The present study has some limitations. We identified the mechanism of the synergistic effect through expression of neuroprotective proteins, but further study is necessary to examine whether the combined treatment increases the level and availability of reduced dopamine. Kim et al. [16] found that acupuncture treatment did not restore the total level of dopamine but increased the DOPAC/DA and HVA/DA ratios, reflecting increased metabolism and release of dopamine. Therefore, the level of striatal dopamine may not necessarily be correlated with improvements in abnormal motor function [48, 49]. However, the possibility that the combined treatment increases the dopamine level should not be ruled out. Therefore, further studies are warranted to elucidate the mechanism how this synergistic effects take place.

To clarify the detailed mechanisms of KD5040, it is necessary to investigate the effects of single herbs and the synergistic effects between or among herbs, because KD5040 is composed of numerous herbs. Previous studies have explored the therapeutic effects and compounds of various herbs. The major element of *Paeonia lactiflora* rhizome is paeoniflorin [26]. It protects PC12 cells from MPP+ and acidic damage through the autophagic pathway [50]. It also improves neurological impairment in striatal 6-OHDA lesion PD rat models [51] and attenuates neuroinflammation and dopaminergic neurodegeneration in adenosine A1 receptor-induced PD mouse models [52]. Geniposide is an active element of *Gardenia jasminoides*. It suppresses *α*-synuclein expression, thereby having neuroprotective effects [53]. It also induces growth factors and reduces apoptosis in the MPTP-induced PD mouse model [54]. Eugenol is extracted from *Eugenia caryophyllata*. It has an antioxidant effect [55], has shown neuroprotective effects in the PD mouse model [56], and inhibits monoamine oxidase (MAO) activity [57]. Other researchers analyzed the compounds of KD5040 using gas chromatography-mass spectrometry (GC-MS) [28]. It will also be necessary to confirm whether KD5040 regulates dopaminergic systems or other therapeutic pathways. Based on these results, we are planning additional studies to investigate the pharmacologic properties of KD5040 based on cell-level tests.

It is necessary to confirm that the effect can be reproduced in other PD models, such as the *α*-synuclein gene mutation mouse model or the specific brain area destruction PD model because this study was limited to the subchronic MPTP-induced PD mouse model. This study used the combined treatment that could be helpful for patients. Further research will be needed to confirm the usefulness and clinical basis of the combined treatment through clinical studies.

Although the present study has some limitations, the findings provide valuable insights about the synergistic effects of the combined treatment. No previous studies have explored the therapeutic effects of the combined treatment. We investigated whether the combined treatment involves synergistic and/or negative effects and found that the combined treatment has significant therapeutic effects without negative effects. Our present study has a meaning of translational research from bed to bench side; it may support the usage of the combined treatments in the clinical field and thereby contribute to finding effective therapies.

## 5. Conclusions

The combined treatment of acupuncture and low-dose KD5040 exhibited a synergistic effect on abnormal motor function and neuroprotection compared with treatment with acupuncture or KD5040 alone. Moreover, the neuroprotective effect of dopaminergic neurons occurred via activation of the PI3K/AKT/GSK3*β* and MEK/ERK pathways. These results may lead to further studies regarding the efficacy of the combined treatment for clinical application.

## Data Availability

The experimental data used to support the findings of this study are available from the corresponding author upon request.

## Supplementary Materials

Supplementary Materials Supplementary Figure 1: Acu and KDL treatment on control mice. Supplementary Figure 2: experiment for confirming cytotoxicity of KD5040.
