# Supplementary material for: Effects of Combined Treatment with Acupuncture and Chunggan Formula in a Mouse Model of Parkinson's Disease
Source: Evid Based Complement Alternat Med. 2019 Nov 21;2019:3612587. doi: 10.1155/2019/3612587 (PMC6907061; doi:10.1155/2019/3612587)
Supplement: Supplementary Materials — Supplementary Figure 1: Acu and KDL treatment on control mice. Supplementary Figure 2: experiment for confirming cytotoxicity of KD5040. [file 3612587.f1.docx]

**Supplementary Figure 1. Acu and KDL treatment on control mice.** To investigate whether Acu or KDL affects to the motor function of the control mice, we treated Acu and KDL to the control mice. Rotarod test was performed on the last day. Three mice groups did not show the significant difference. Through this result, we could know that Acu and KDL did not affect to the control mice.


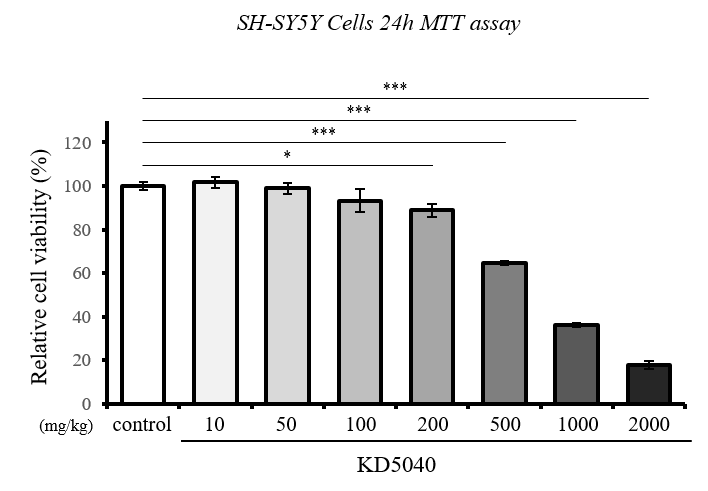


**Supplementary Figure 2. Experiment for confirming cytotoxicity of KD5040.** We investigated cytotoxicity of KD5040 on different doses. When the doses of KD5040 were below 100 mg/kg, the cell viability were not different compared with control. However, at 200 mg/kg of KD5040, the cell viability showed a significant difference. The cell viability sharply decreased when the dose of KD5040 was more than 500 mg/kg.
